# Sparse phenotyping for wheat grain yield enabled by multiomics prediction

**DOI:** 10.1002/tpg2.70298

**Published:** 2026-09-06

**Authors:** Guillermo Gerard, Velu Govindan, Susanne Dreisigacker, Keith Gardner, Flavio Breseghello, Leonardo Crespo‐Herrera, Matthew Reynolds, Jose Crossa, Paolo Vitale

**Affiliations:** ^1^ International Maize and Wheat Improvement Center (CIMMYT) El Batán Mexico; ^2^ Colegio de Postgraduados, Campus Montecillo, Carretera México‐Texcoco Texcoco Mexico

## Abstract

Grain yield is a central target in wheat breeding, yet accurately predicting it remains challenging because it depends on many genes and responds strongly to environmental variation. Genomic selection (GS) has improved breeding efficiency by enabling genome‐based prediction of genetic merit, but predictability (PA) for grain yield is often limited under stress environments. At the same time, advances in high‐throughput phenotyping (HTP) using unmanned aerial vehicles (UAVs) provide phenomic data that capture environment‐responsive plant performance and may complement genomic information. In this study, we evaluated genomic and phenomic models for predicting grain yield in elite bread wheat lines across irrigated, drought, and heat‐stress environments. Using a sparse phenotyping framework, we compared parametric and non‐parametric models. PA was evaluated within environments and under cross‐environment sparse phenotyping scenarios. Genomic models provided a stable baseline and enabled effective information sharing across environments when phenotypic data were incomplete. Phenomics‐only models captured environment‐specific plant responses but were more sensitive to environmental context. Multiomics models that integrated genomic and phenomic information consistently achieved the highest PA, with the largest gains observed under stress conditions. Overall, our results demonstrate that integrating genomics and UAV‐based phenomics within sparse phenotyping designs offers a practical and scalable approach to improve grain yield prediction in wheat.

## Plain Language Summary

Wheat grain yield predictability is often scarce because of the complexity of the trait. This poses a challenge for breeders and quantitative genetics implementing genomic selection in their breeding programs. In this research, we explored a multiomics prediction framework within a sparse‐phenotyping design to predict grain yield in wheat by jointly using genomics and unmanned aerial vehicle (UAV)‐based high‐throughput phenotyping (HTP) data in a real‐world validation scenario. We found that combining the two data sources improved the predictability of grain yield compared with using either genomics or phenomics alone. These results highlight the added value of UAV‐based HTP for supporting more accurate and efficient selection decisions in wheat breeding programs.

**Abbreviations**
BDRT: beds under drought
BEHT: Beds early heat stress
BLUE: best linear unbiased estimate
CIMMYT: International Maize and Wheat Improvement Center
G × E: genotype × environment interaction
G‐P‐RKHS‐M1: genomic and phenomic RKHS using method 1
G‐P‐RKHS‐M2: genomic and phenomic RKHS using method 2
GBLUP: genomic best linear unbiased prediction
GP: genomic prediction
GRM: genomic relationship matrix
GS: genomic selection
GY: grain yield
HTP: high‐throughput phenotyping
NIR: near‐infrared
PA: predictability
PBLUP: phenomic best linear unbiased prediction
P‐GBLUP‐M1: phenomic and genomic BLUP using method 1
P‐GBLUP‐M2: phenomic and genomic BLUP using method 2
P‐RKHS: reproducing kernel Hilbert space using phenomics
PRM: phenomic relationship matrix
SNP: single‐nucleotide polymorphism
RKHS: reproducing kernel Hilbert space using genomics
SREG: site regression analysis
UAV: unmanned aerial vehicle

## 1 INTRODUCTION

Improving grain yield (GY) remains a central objective in wheat (*Triticum aestivum* L.) breeding programs worldwide, yet it remains one of the most difficult traits to predict and improve efficiently. GY is a complex quantitative trait controlled by many loci of small effect and strongly influenced by environmental variability and management practices. As a consequence, genotype × environment (G × E) interactions are significant, particularly in breeding pipelines that operate across contrasting water and temperature regimes, such as optimal irrigation, drought stress, and heat stress. These interactions complicate selection decisions and often limit the stability of genetic gains across environments.

Genomic selection (GS) has transformed modern plant breeding by enabling the prediction of genetic merit using genome‐wide molecular markers (Meuwissen et al., 2001). Since its introduction, GS has been widely adopted in wheat and other crops, demonstrating clear advantages over traditional phenotypic selection for complex traits such as GY (Crossa et al., 2010; Hickey et al., 2017). By shortening breeding cycles and increasing selection intensity, GS has become a cornerstone of modern breeding programs. Nevertheless, predictability (PA) for GY often remains moderate, particularly when predictions are required across environments and years or when phenotypic data are incomplete (Burgueño et al., 2012; Heslot et al., 2014; Vitale et al., 2025).

To address these limitations, multienvironment genomic prediction models that explicitly incorporate G × E interactions have been developed. Reaction‐norm and site‐regression models have shown that borrowing information across correlated environments can substantially improve predictive performance relative to single‐environment analyses (Burgueño et al., 2012; Jarquín et al., 2014). Recently, multienvironment GP models have been extensively implemented in wheat breeding (García‐Barrios et al., 2024; Gill et al., 2021; Puglisi et al., 2026). Kernel‐based approaches, such as reproducing kernel Hilbert space (RKHS) regression, further extend these models by capturing nonlinear genotype responses across environments, which are common for GY under stress conditions (Cuevas et al., 2016; Gianola & Van Kaam, 2008).

A major practical constraint in breeding programs is the cost and logistics associated with collecting high‐quality phenotypic data across all genotypes and environments. Large populations and extensive multienvironment testing often result in unbalanced datasets, motivating the use of sparse phenotyping (or sparse testing) strategies. Under sparse testing, only a subset of genotypes is phenotyped in each environment, while missing observations are predicted using genomic information and cross‐environment correlations. Several studies have demonstrated that genome‐enabled sparse testing can substantially improve testing efficiency without sacrificing PA when appropriately designed (Crespo‐Herrera et al., 2021a; Jarquin et al., 2020; Montesinos‐López et al., 2023, 2025).

In parallel, advances in high‐throughput phenotyping (HTP) technologies have created new opportunities to rapidly and non‐destructively characterize plant performance. Unmanned aerial vehicles (UAVs) equipped with multispectral and hyperspectral sensors enable repeated measurements of canopy reflectance throughout the growing season, capturing information related to biomass accumulation, canopy structure, pigment composition, and stress responses. In wheat, spectral reflectance has been shown to be associated with GY and stress tolerance, particularly under drought and heat stress conditions (Araus et al., 2018).

Phenomics data can be incorporated into prediction models in several ways. One influential approach is to construct phenomic relationship matrices from spectral reflectance data, analogous to genomic relationship matrices, thereby capturing genetically structured similarity among genotypes based on phenomic traits. Krause et al. (2019) demonstrated that hyperspectral reflectance‐derived relationship matrices can be effectively integrated into genomic prediction models to improve GY prediction in wheat. More broadly, phenomic prediction has emerged as a complementary strategy to genomic prediction, with several studies reporting competitive or synergistic performance depending on trait architecture, environment, and data availability (Brault et al., 2022; Galán et al., 2020; Winn et al., 2023).

Despite these advances, important challenges remain when integrating phenomics into breeding prediction pipelines. Phenomic data are typically high‐dimensional, temporally structured, and environment‐dependent, and their relationships with GY may vary across developmental stages and stress scenarios. Moreover, under sparse phenotyping designs, phenomic measurements may also be missing for subsets of genotypes and environments, requiring strategies to either summarize phenomic information across environments or predict missing spectral values prior to model fitting (Krause et al., 2019; Robert et al., 2022).

In large wheat breeding programs operating across diverse target environments, an improved understanding of how genomics, phenomics, and their integration perform under sparse phenotyping designs is critical for optimizing prediction pipelines. In particular, evaluating alternative strategies for constructing phenomic predictors across environments and assessing the performance of multiomic models that combine genomic and phenomic information can provide practical guidance for breeders.

The objectives of this study were therefore to (i) evaluate genomic prediction performance for GY within environments and under sparse phenotyping design; (ii) assess phenomics‐based prediction using UAV multispectral data through alternative cross‐environment strategies; and (iii) compare genomic, phenomic, and multiomic models across contrasting water and heat stress environments. Using a large set of elite bread wheat lines evaluated under representative International Maize and Wheat Improvement Center (CIMMYT) selection environments, this study provides empirical insights into how genomics and UAV‐based phenomics can be jointly exploited to enhance GY prediction when phenotyping resources are limited.

## 2 MATERIALS AND METHODS

### 2.1 Plant material and genotyping

A total of 1029 new elite bread wheat lines were tested in the CIMMYT experimental field in Ciudad Obregón, Sonora, Mexico, during the crop season 2022–2023. These lines were part of the stage 2 yield testing, also known as the Elite Yield Trial. These are composed lines from the F6 generation. The lines were planted in incomplete block designs with 19 sub‐trials with a block size of five plots. Each sub‐trial included 54 candidate lines and six checks in two replications with 12 blocks. The six checks were repeated for each sub‐trial. The tested lines were evaluated in each of the following simulated environments (Crespo‐Herrera et al., 2021b):

- Beds intermediate drought (beds planting system with two irrigations [B2IR]): this trial was grown on raised beds with partial irrigation (about 260 mm of available water in two irrigations), which was also characterized by an optimal sowing date (late November to mid‐December).
- Beds optimal environment (beds planting system with five irrigations [B5IR]): the trial was planted on raised beds with optimal irrigation (about 500 mm of available water in five irrigations) and an optimal sowing date.
- Beds under drought (BDRT): this trial was performed on raised beds with severe drought conditions (about 180 mm of available water) and an optimal sowing date.
- Beds early heat stress (BEHT): the trial was sown in October (earlier than the optimal sowing period) to expose the candidate varieties to initial heat stress, and this was also carried out with optimal irrigation.

At maturity, whole plots were harvested to assess GY. A plot area of 4.48 m2 (2.8 × 1.6) was used in beds to estimate GY per plot. Finally, GY was adjusted to a moisture content of 12%.

The DNA was isolated from dried leaf tissue collected from five plants per line using a modified CTAB extraction protocol, following CIMMYT laboratory guidelines (Dreisigacker et al., 2016). Genotyping produced a dataset of 18,239 single‐nucleotide polymorphisms (SNPs). Genotyping‐by‐sequencing libraries and SNP calling followed the wheat GBS workflow described by Poland et al. (2012), with library preparation performed through the CIMMYT/Kansas State University pipeline. Markers with minor allele frequency <0.05 and those with >50% missing data were removed. Remaining missing genotypes were imputed using BEAGLE v5.0. After these steps, the HapMap output was converted to a numeric genotype matrix for downstream analyses and compatibility with genomic prediction software.

### 2.2 Flight procedures

Five UAV flight dates were used to acquire images for each of the simulated environments, from the vegetative stage to maturity. Because these flight dates were calendar‐based rather than explicitly aligned by thermal time or exact physiological stage, spectral measurements across environments may partly reflect differences in crop development, senescence, and stress progression. A DJI Matrice 100 equipped with a MicaSense RedEdge‐M multispectral camera (MicaSense, Inc.) was used for image acquisition. The multispectral sensor collected five spectral bands with the following nominal center wavelengths and full width at half maximum: blue (475 nm, 20 nm), green (560 nm, 20 nm), red (668 nm, 10 nm), red edge (717 nm, 10 nm), and near‐infrared (NIR) (840 nm, 40 nm). To standardize operations across flights, all UAV missions were pre‐programmed using the UAVmissionplanner platform (https://uavmissionplanner.netlify.app/) in combination with the Litchi flight planning application (https://flylitchi.com/hub). Ground control points were also used. Finally, before each flight, radiometric calibration was performed using the MicaSense reflectance panel. The orthomosaic was built using the software Pix4Dmapper (Pix4D SA). The company Hiphen – “Agricultural digital solution” performed quality control and wavelength extraction for each plot (https://www.hiphen‐plant.com).

### 2.3 Statistical analyses

Best linear unbiased estimates (BLUEs) were calculated for each line in the R environment using the software ASREML‐R (https://vsni.co.uk/software/asreml‐r/) using the following linear mixed model:

(1) $$y_{ikjl}=\mu +g_{i}+t_{k}+r_{j\left(k\right)}+b_{l\left(j\left(k\right)\right)}+\varepsilon_{ikjl},$$

where the $y_{ikjl}$ is the plot collected trait (GY or wavelength per flight), μ is the model intercept, $g_{i}$ is the *i*th line effect assumed to be fixed, $t_{k}$ is the random effect of the *k*th sub‐trial, $r_{j(k)}$ is the *j*th replication effect within the *k*th sub‐trial, $b_{l(j(k))}$ is the *l*th block effect within the *j*th replication and the *k*th sub‐trial, and $\varepsilon_{ikjl}$ is the error effect assumed to be independent and identically distributed $\varepsilon_{\mathrm{ikjl}}∼N\left(0,\sigma_{\varepsilon }^{2}\right)$. By using the same model with the exception of setting the line effect ($g_{i}$) as random, components of variance were estimated and the broad‐sense heritability ($H^{2}$) was calculated using the following equation:

(2) $$H^{2}=\frac{\sigma_{g}^{2}}{\sigma_{g}^{2}+\frac{\sigma_{\varepsilon }^{2}}{r}}\;,$$

where $\sigma_{g}^{2}$ is the genotypic variance, $\sigma_{\varepsilon }^{2}$ is the error variance, and r is the number of replications (Piepho & Möhring, 2007). Additionally, we also estimated an adjusted mean for each wavelength across flight dates. To perform this, additional factors such as $f_{s}$ (random effect of the *s*th flight date) and $gf_{is}$ (the random interaction effect between flight date and the genotype) were added to Equation (1). Finally, G × E analysis using site regression analysis (SREG) was performed, starting with the GY‐ and wavelength‐adjusted means. This last analysis was performed using the software GEA‐R (https://hdl.handle.net/11529/10203). Model specifications can be found in the software's manual.

### 2.4 Prediction models

Several models were performed within the environment approach, using sparse testing strategies, through the use of genomics, phenomics, and both omics. Here, there is a model summary:

- Two reference models based only on genomics data were used in the within‐environment strategy using fivefold cross‐validation: genomic best linear unbiased predictors (GBLUP) and RKHS.
- A phenotypic baseline (PB) model was run using only phenotypic information in a sparse phenotyping or CV2 validation design.
- The same models, GBLUP and RKHS, were also run using a cross‐environment approach in a sparse phenotyping design validation or CV2 using only genomics information.
- The same models and same sparse phenotyping validation were also performed using phenomic data through a phenomic‐BLUP (PBLUP), only in two methods:
  ○ M1: Computing wavelengths BLUE across the environments using a linear mixed model and subsequently building a cross‐environment phenomic relationship matrix (PRM), which was used as a predictor. This included both PBLUP‐M1 and P‐RKHS‐M1.
  ○ M2: Using markers to predict the missing wavelength for each environment, building a PRM for each environment separately, and subsequently using a merged PRM as a predictor. This included both PBLUP‐M2 and P‐RKHS‐M2.
- Multiomic models combining genomics and phenomics were also performed using a sparse phenotyping validation following M1 and M2 for the phenomic‐based predictors. This included P‐GBLUP‐M1, P‐GBLUP‐M2, G‐P‐RKHS‐M1, and G‐P‐RKHS‐M2.
- The multiomic approach was also performed using the hybrid kernel matrix under the M1 (Cuevas et al., 2025).

Hereafter, the model description and the validations were reported.

### 2.5 Within‐environment prediction

We first ran two reference models, GBLUP and RKHS, in each simulated environment using only genomics data. The GBLUP model was performed as follows:

(3) $$y_{i}=\mu +g_{i}+\varepsilon_{i},$$

where $y_{i}$ represents the GY BLUE for the *i*th genotype, μ is the intercept, $g_{i}$, i=1,…,J represents the genomic breeding value for the *i*th genotype, and $\varepsilon_{i}$ is the error assumed to be independent and normally distributed with mean 0 and variance $\sigma_{\varepsilon }^{2}$. It is assumed that $g={\left(g_{1},\text{…},g_{J}\right)}^{T}\;∼N_{J}\left(0,G\sigma_{G}^{2}\right)$, where G is the genomic relationship matrix (GRM) built from the SNP matrix, and $\sigma_{G}^{2}$ is the additive genetic variance (VanRaden, 2008).

Subsequently, RKHS with kernel averaging was applied as follows:

$$y_{i}=\mu +\sum_{l=1}^{L}u_{li}+\varepsilon_{i},\;\left(4\right)$$

where $y_{i}$, μ, and $\varepsilon_{i}$ have been reported in Equation (3), $u_{li}$ is the additive genetic effect of the *i*th genotype corresponding to the *l*th Gaussian kernel, and $u_{l}=\left(u_{l1},\cdots ,\;u_{\mathrm{lJ}}\right)∼\;N_{J}\left(0,\;K_{l}\sigma_{u_{l}}^{2}\right)$ is the additive genetic effect with $K_{l}$ corresponding to the Gaussian reproducing kernel evaluated at *l*th of the bandwidth parameters and $\sigma_{u_{l}}^{2}$ is the additive genetic variance as reported in the package BGLR (Pérez & De Los Campos, 2014). Both GBLUP and RKHS models were run as single chains of 12,000 iterations, with the first 5000 discarded as burn‐in to ensure convergence. The analysis was carried out using the BGLR library (Bayesian Generalized Linear Regression) (Pérez & De Los Campos, 2014).

### 2.6 Sparse phenotyping models

#### 2.6.1 Phenotypic baseline

A phenotypic data‐only model (PB) was tested in a separate phenotypic group for CV2‐like validation, in which some individuals were observed in some environments but not in others. The PB models were as follows:

(5) $$y_{ji}=\mu +E_{j}+L_{i}+\varepsilon_{ji},$$

where $y_{ji}$ is the vector of BLUEs for GY, μ is the intercept, $E_{j}$ is the random effect of the *j*th simulated environment, and $L_{i}$ is the random effect of the *i*th line, assuming $L_{i}\;∼\;NIID\left(0,\sigma_{L}^{2}\right)$, and $\varepsilon_{ji}$ is the error assumed to be independent and normally distributed with mean 0 and variance $\sigma_{\varepsilon }^{2}$.

#### 2.6.2 Only genomics

The sparse phenotyping validation followed a CV2‐type design. It was performed using the following GBLUP model, which incorporated the G × E interaction:

(6) $$y_{ji}=\mu +E_{j}+L_{i}+g_{i}+{\left(gE\right)}_{ij}+\varepsilon_{ji},$$

where $y_{ji}$, μ, $E_{j}$, $L_{i}$, and $\varepsilon_{ji}$ were described in model 5; $g\;=(g_{1},\text{…},g_{J})$ is the vector of the genetic effects, which presents a normal distribution $g\;∼\;N(0,\left(Z_{g}G{Z^{\prime }}_{g}\right)\sigma_{g}^{2})$, where $Z_{g}$ is the incidence matrix linking the observations with the genomics; G is the GRM; and $\sigma_{g}^{2}$ is the variance associated with g. $gE_{ij}$ is the G × E interaction term assuming $gE=\left\{gE_{ij}\right\}\;\;∼\;N\left(0,\left(Z_{g}G{Z^{\prime }}_{g}\right)\#\left(Z_{E}{Z^{\prime }}_{E}\right)\sigma_{gE}^{2}\right),$ where # corresponds to the Hadamard product; $Z_{E}$ is the incidence matrix for the environments; and $\sigma_{gE}^{2}$ is the error associated with the interaction term.

Additionally, the G × E interaction component was also integrated in the RKHS model as follows:

$$y_{ji}=\mu +E_{j}+L_{i}+\sum_{l=1}^{L}u_{l,i}+\sum_{l=1}^{L}{\left(uE\right)}_{l,ij}+\varepsilon_{ji},\;\left(7\right)$$

where the terms $y_{ji}$, μ, $E_{j}$, $L_{i}$, and $\varepsilon_{ji}$ were reported in model 5, $u_{l}=\left(u_{l1},\text{…},u_{lJ}\right)$ assuming $u\;∼\;N(0,\sum_{l=1}^{L}\left(Z_{g}K_{l}{Z^{\prime }}_{g}\right)\sigma_{ul}^{2})$ where $K_{l}$ are the kernel‐based relationship matrices computed starting from molecular markers information, and $uE=\left\{uE_{l,ij}\right\}\;\;∼\;N(0,\;\left(\sum_{l=1}^{L}\left(Z_{g}K_{l}{Z^{\prime }}_{g}\right)\#\left(Z_{E}{Z^{\prime }}_{E}\right)\sigma_{uE}^{2}\right)$ where $\sigma_{uE}^{2}$ is the error associated with the interaction component, and all elements have already been described.

#### 2.6.3 Only phenomics

Additionally, only phenomics‐based models were implemented in a sparse phenotyping design validation. The PBLUP was equivalent to GBLUP (Equation 6), with the exception that in the PBLUP, the g genomic effects are now the phenomic effects $h\;=(h_{1},\text{…},h_{J})$ assuming $h\;∼\;N(0,\left(Z_{h}H{Z^{\prime }}_{h}\right)\sigma_{h}^{2})$ where H corresponds to the PRM and was computed as follows: $H\;=\frac{{\mathrm{SS}}^{\prime }}{n}$, where S is the scaled and centered matrix made by the wavelengths and n is the number of wavelengths (Krause et al., 2019). Additionally, an interaction term $hE_{ij}$ as the phenomics‐by‐environment interaction (P×E) term was included, assuming $hE=\left\{hE_{ij}\right\}\;\;∼\;N\left(0,\left(Z_{h}H{Z^{\prime }}_{h}\right)\#\left(Z_{E}{Z^{\prime }}_{E}\right)\sigma_{hE}^{2}\right)$ where #, $Z_{h}$, and $Z_{E}$ were previously defined, and $\sigma_{hE}^{2}$ is the error associated with the interaction term.

Similarly, the H‐RKHS showed the same model structure as the RKHS, with the exception of the $\sum_{l=1}^{L}u_{l,i}\;\mathrm{and}\;\sum_{l=1}^{L}{\left(uE\right)}_{l,ij}$ that were replaced with $\sum_{l=1}^{L}p_{l,i}\;\mathrm{and}\;\sum_{l=1}^{L}{\left(pE\right)}_{l,ij}$ where $p_{l}=\left(p_{l1},\text{…},p_{lJ}\right)$ assuming $p\;∼\;N(0,\sum_{l=1}^{L}\left(Z_{p}K_{l}{Z^{\prime }}_{p}\right)\sigma_{pl}^{2})$ where $K_{l}$ are the kernel‐based relationship matrices computed using the phenomics‐derived information, and $pE=\left\{pE_{l,ij}\right\}\;\;∼\;N(0,\;\left(\sum_{l=1}^{L}\left(Z_{p}K_{l}{Z^{\prime }}_{p}\right)\#\left(Z_{E}{Z^{\prime }}_{E}\right)\sigma_{pE}^{2}\right)$ where $\sigma_{pE}^{2}$ is the error associated with the interaction component. It is essential to highlight that for PBLUP and P‐RKHS, the wavelengths were computed using two methods: M1 and M2.

In M1, the following mixed linear model was implemented to estimate wavelengths BLUE across the selection environments: $y_{ij}=\mu +E_{j}+L_{i}+{\left(LE\right)}_{ji}+\varepsilon_{ij}$ where $y_{ij}$ is the BLUE of the wavelength at the *i*th line in the *j*th environment, μ is the model intercept, $E_{j}$ is the random effect of the *j*th environment, $L_{i}$ is the fixed effect of the *i*th line, ${\left(LE\right)}_{ji}$ is the G × E interaction component, and $\varepsilon_{ij}$ is the error term. Subsequently, the BLUEs of the wavelengths were used to compute H, which was used as a predictor in the main prediction model.

In M2, a GBLUP model was used to predict the missing values for each wavelength. In model 3, y now corresponds to the BLUE vector for each wavelength in each environment. Therefore, for each *j*th environment, $H_{j}$ was computed using the observed and the predicted values for each wavelength. Subsequently, a combined PRM $\left(H_{\mathrm{cb}}\right)$ was computed according to Robert et al. (2022) as follows: $H_{\mathrm{cb}}=\frac{S_{\mathrm{cb}}S\prime_{\mathrm{cb}}}{n}$, where $S_{\mathrm{cb}}$ is the centered and scaled matrix of wavelengths, adding one next to the other from environment 1 to the *j*th. For the M1 method, the wavelengths were computed using the same modeling approach for PBLUP and P‐RKHS; in the M2 method, missing wavelengths were predicted using GBLUP (model 6) and RKHS model (7) for PBLUP and P‐RKHS, respectively.

#### 2.6.4 Multiomic models

We extend the model reported in Equation (5) to both omics: genomics and phenomics. In this scenario, the genetic effects g used in the GBLUP were used alongside the phenomic effects h used in the PBLUP. For the multiomic prediction, the following P‐GBLUP model was used:

(8) $$y_{ji}=\mu +E_{j}+L_{i}+g_{i}+h_{i}+{\left(gE\right)}_{ij}+{\left(hE\right)}_{ij}+\varepsilon_{ji},$$

where all the terms were previously defined. In this P‐GBLUP model, both GRM and PRM were used. As for the only phenomics‐based models, the P‐GBLUP was performed in both M1 and M2. Using the same philosophy, a G‐P‐RKHS model based on kernel matrices computed from both molecular markers and wavelengths was also performed as follows:

(9) $$\begin{array}{ccc} y_{ji} & = & \mu +E_{j}+L_{i}+\sum_{l=1}^{L}u_{l,i}+\sum_{l=1}^{L}p_{l,i}\\ & & +\;\sum_{l=1}^{L}{\left(uE\right)}_{l,ij}+\sum_{l=1}^{L}{\left(pE\right)}_{l,ij}+\varepsilon_{ji},\; \end{array}$$

where all the model elements were previously described. Also, the G‐P‐RKHS was performed in M1 and M2.

Additionally, we implemented a hybrid kernel method following the framework introduced by Cuevas et al. (2025). The $C_{b}$ and $P_{b}$ matrices were included in the prediction model alongside the kernel matrices computed from markers and wavelengths. These were derived from the upper and lower triangles, respectively, of the matrix calculated from the product between the kernel matrices derived from the markers $\left(Kg_{b}\right)$ and kernel matrices derived from the wavelengths $\left(Kh_{b}\right)$ for each *b*th bandwidth parameter. For additional details, see Cuevas et al. (2025).

#### 2.6.5 Validation strategies

For within‐environment validation, a fivefold cross‐validation was performed 10 times. Model PA was estimated as the correlation between the observed and predicted values. The final PA result was averaged across all runs.

**FIGURE 1:**
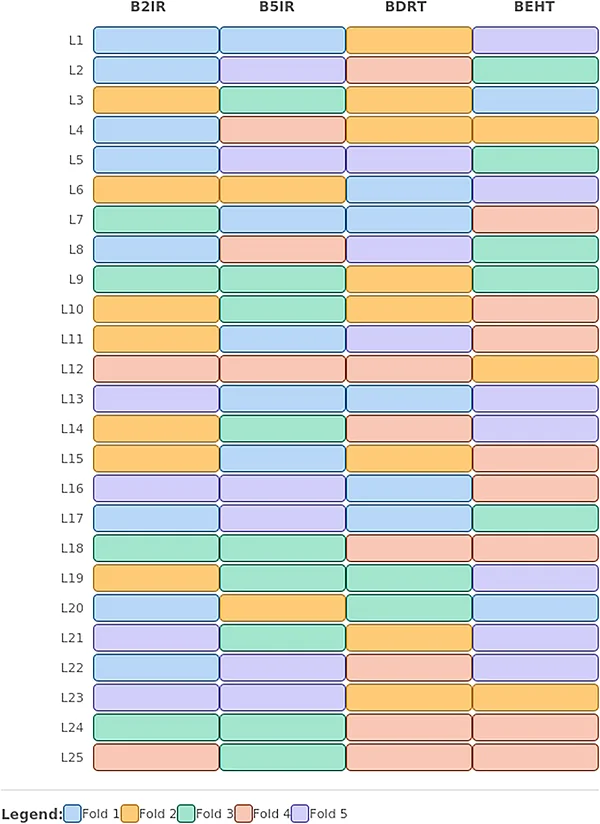
Sparse phenotyping (CV2) validation example plot with the allocation of 25 lines from four environments in five random folds.

For the sparse phenotyping design validation, a 10‐cycle fivefold CV2 was implemented. For more details on CV2 validation, see Burgueño et al. (2012) and Jarquín et al. (2017). This validation strategy aims to emulate a sparse phenotyping scenario in which lines were tested in some environments but not in others. The number of missing values was maintained fixed across the environments. Figure 1 displays an example of lines × environment allocation in five different folds.

It is worth noting that PA was assessed using a Pearson correlation between the BLUEs and the genomic estimated breeding values for the lines within the target fold in each environment. Subsequently, the average PA from each fold in each environment was used as the final PA value. It is essential to highlight that when phenomics was modeled, the lines in each fold that were masked for GY were also masked for wavelengths, attempting to emulate a real‐world scenario in which breeders do not have information on the target lines in the target environments. Subsequently, M1 and M2 were implemented to address missing wavelengths across environments.

## 3 RESULTS

### 3.1 Statistical analyses

Descriptive statistics, component of variance, and heritability for GY are reported in Table 1 for each simulated environment. BLUEs ranged from 3.16 t/ha in BDRT to 9.75 t/ha in B5IR. Heritability ranged from 0.45 to 0.67 in B5IR and BEHT, respectively. The same statistical parameters were also reported for each wavelength, environment, and flight date (Table S1). Interestingly, heritability was high across all wavelengths, reaching 0.92 for blue in B5IR on the first flight date (February 15, 2023).

**TABLE 1:** Best linear unbiased estimates (BLUEs) in t/ha, descriptive statistics, components of variance, and heritability for grain yield in different simulated environments.

Environment	Min	Mean	Max	SD	CV	Vg	Ve	H2
B2IR	4.58	5.84	6.84	0.31	5.27	0.04	0.08	0.50
B5IR	5.72	8.10	9.75	0.42	5.23	0.07	0.17	0.45
BDRT	3.16	4.01	4.80	0.23	5.82	0.03	0.04	0.50
BEHT	5.94	7.89	9.16	0.45	5.76	0.13	0.11	0.67

Abbreviations: CV, coefficient of variation; H^2^, heritability; SD, standard deviation; Ve, error variance; Vg, genetic variance.

**FIGURE 2:**
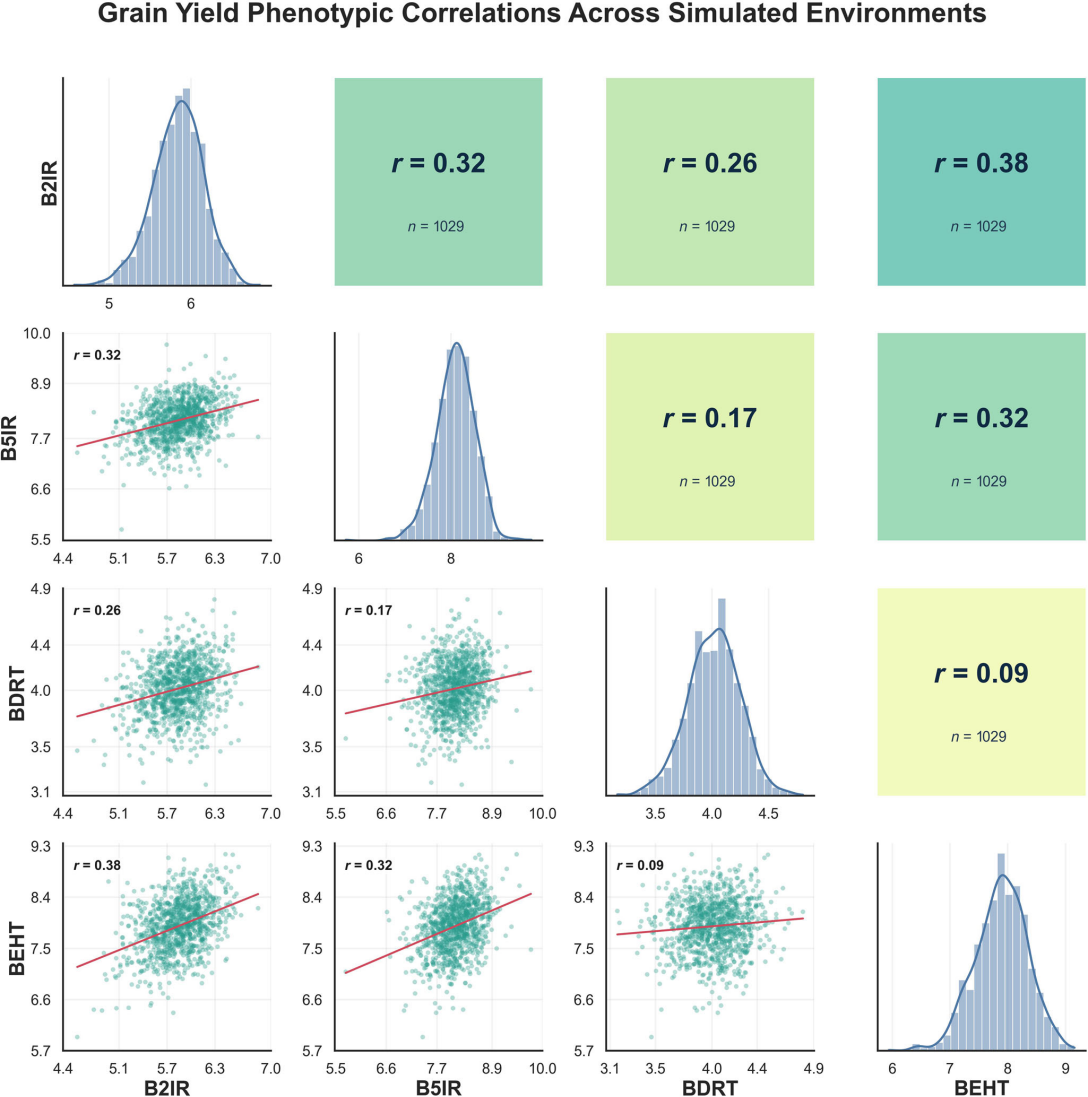
Pearson's correlations for grain yield using best linear unbiased estimates (BLUEs) across the four simulated environments.

Pearson's correlations for grain yield (BLUEs) across the four simulated environments are reported in Figure 2. Interestingly, B2IR and BEHT showed the highest correlation (0.38). By contrast, the lowest correlation (0.09) was observed between the BDRT and BEHT simulated environments.

**FIGURE 3:**
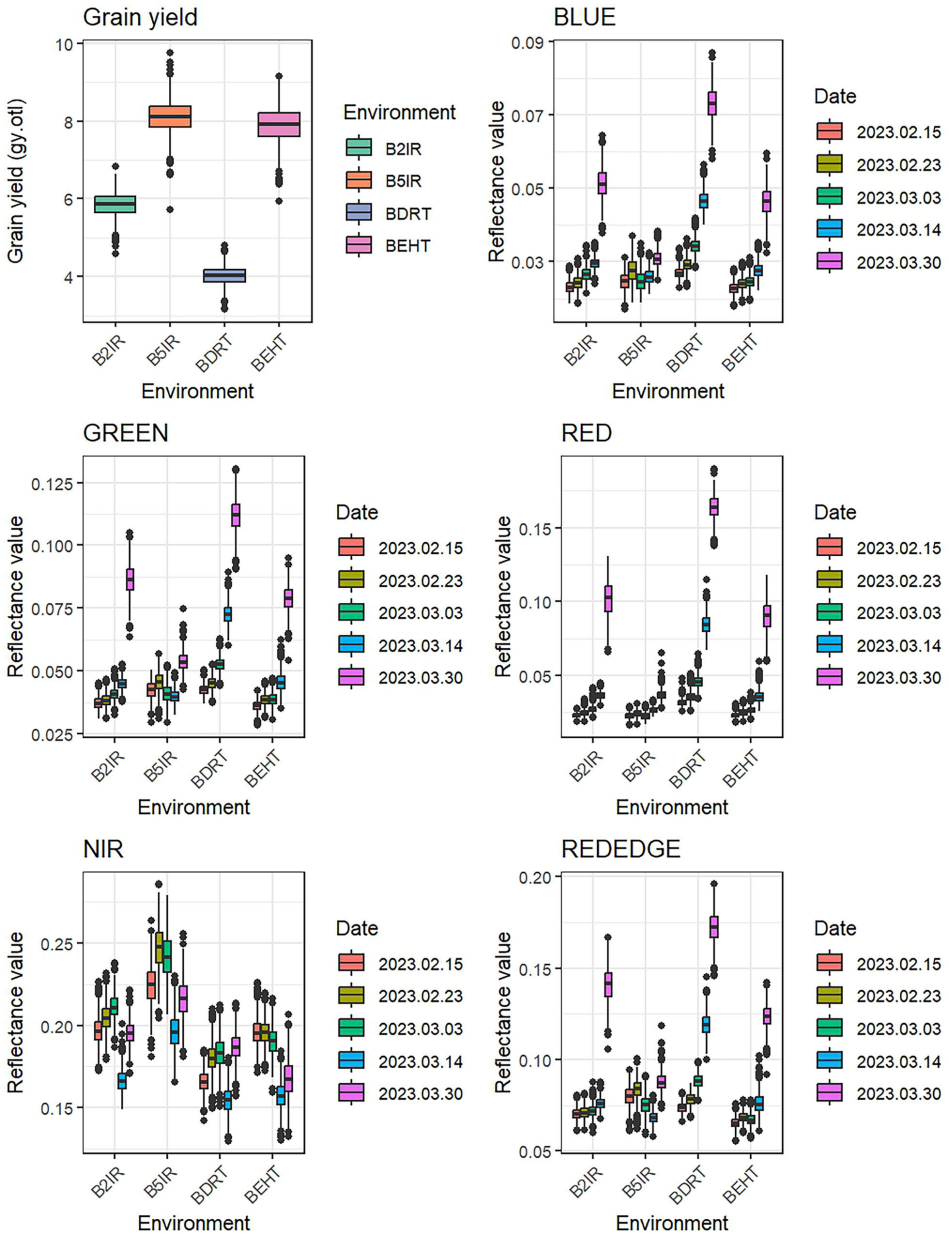
Best linear unbiased estimates (BLUEs) range across environments and flight dates.

Trait distribution was reported in Figure 3. For GY, the environments B5IR and BEHT outperformed the B2IR and the BDRT. Reflectance patterns varied across wavelengths and flight dates. In drought‐stressed environments, higher reflectance values at later flight dates likely reflected advanced crop development, senescence, and stress progression relative to better watered environments. Therefore, spectral differences across environments should be interpreted jointly with crop developmental stage, particularly because flights were conducted on common calendar dates rather than aligned by thermal time.

**FIGURE 4:**
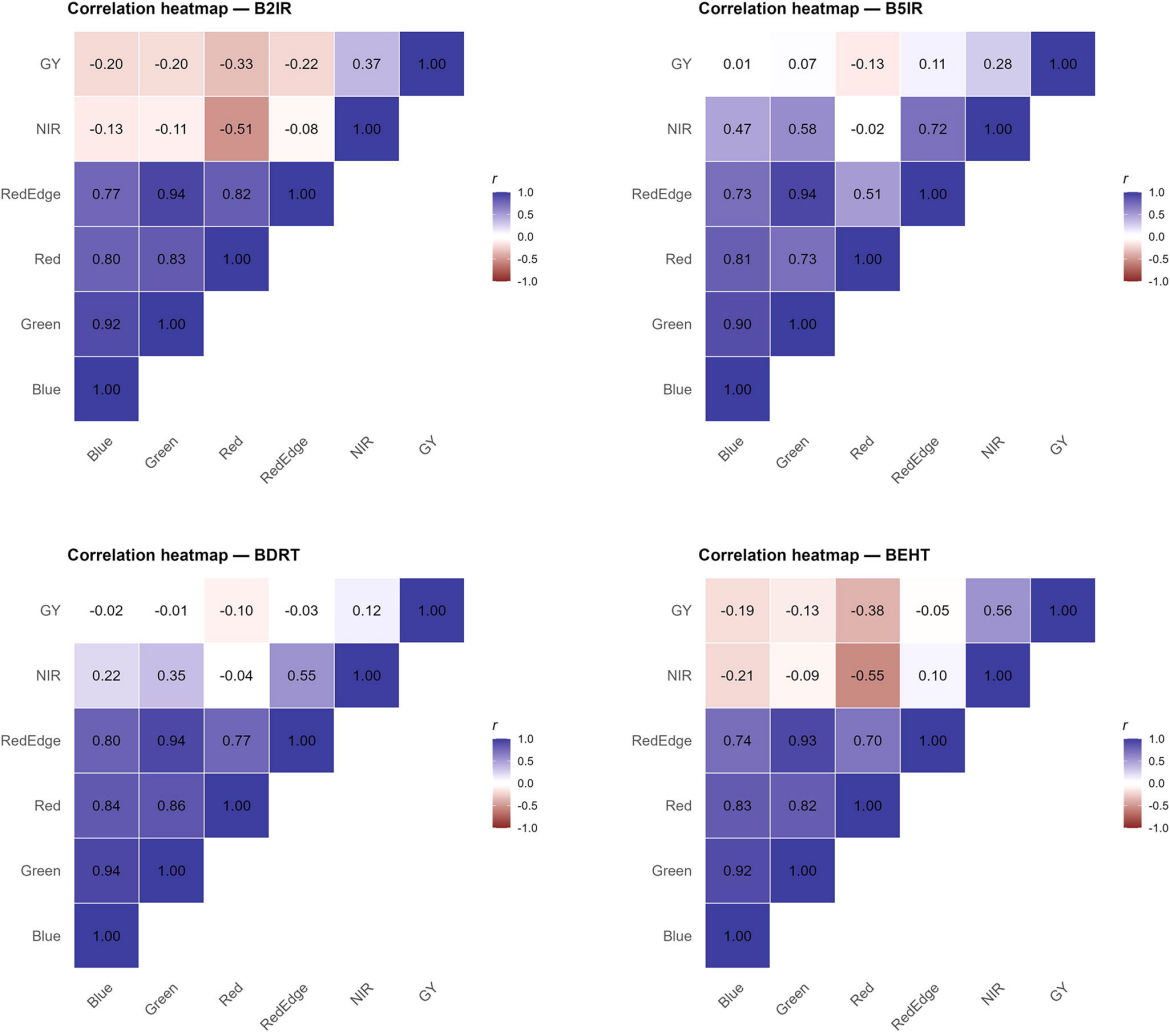
Pearson's correlations among variables for each environment.

BLUEs across flight dates were also used to calculate Pearson's correlations among variables for each environment (Figure 4). Interestingly, wavelengths showed negative correlations with GY in B2IR and BEHT, except for NIR, which was positively correlated with GY (0.37 and 0.56 in B2IR and BEHT, respectively). By contrast, in the remaining environments (B5IR and BDRT), wavelengths showed only a weak correlation with GY. Wavelengths showed high positive correlations among them, with the exception of NIR in the environment B2IR and BEHT. Additionally, the correlations of the BLUEs among the wavelengths for each flight and the GY for each environment are reported in Figures S1–S4.

**FIGURE 5:**
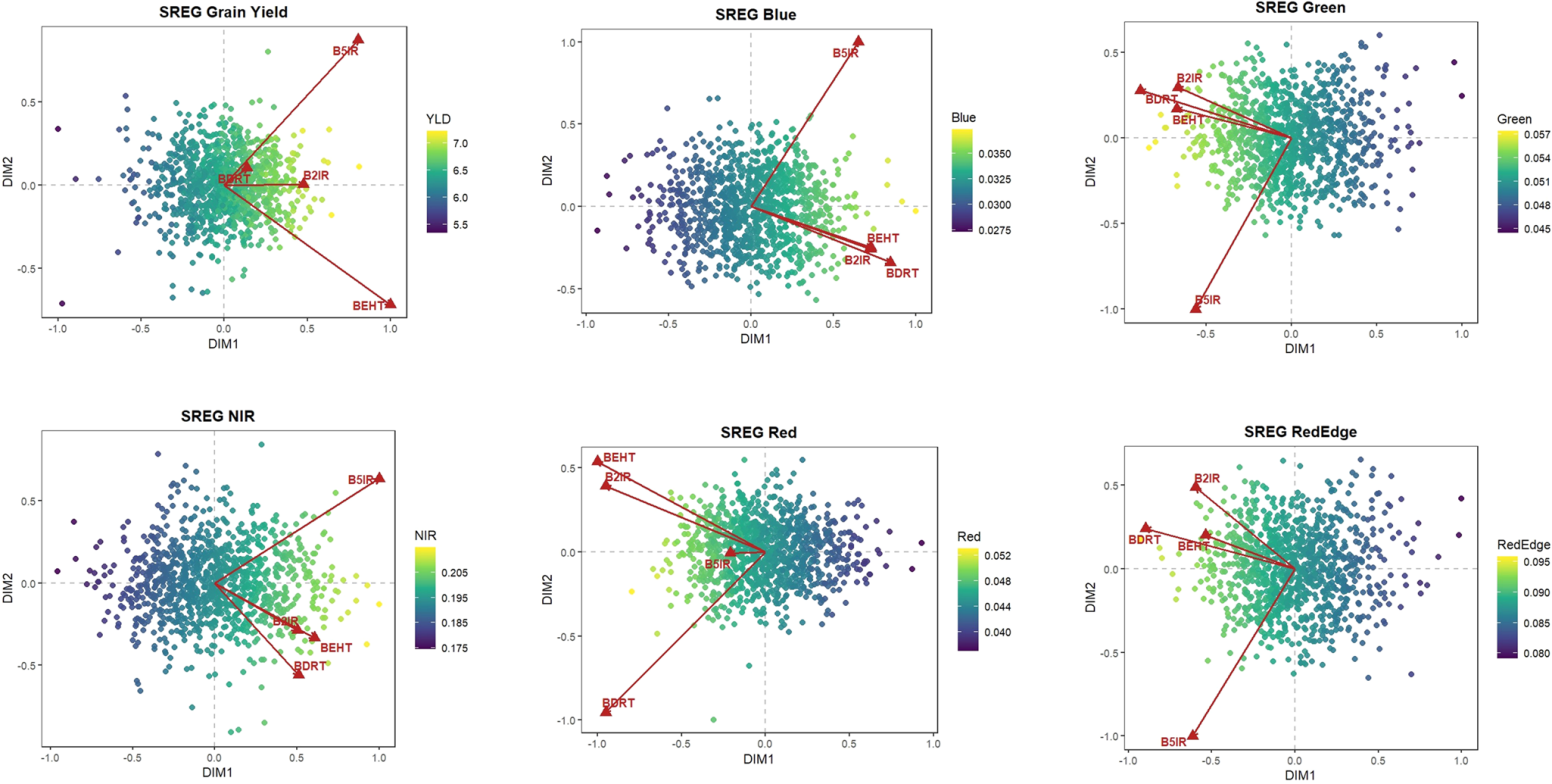
Regression analysis (SREG) for each variable under investigation.

According to the SREG analyses and reported in Figure 5, all variables showed G × E interaction. Regarding GY, the biplot clearly separated the B5IR from the BEHT. Interestingly, all the wavelengths showed very similar patterns. Indeed, all biplots clearly separated B5IR from the other simulated environments, except at the red wavelengths, where BDRT instead was separated from the other environments.

### 3.2 Prediction outcomes

**FIGURE 6:**
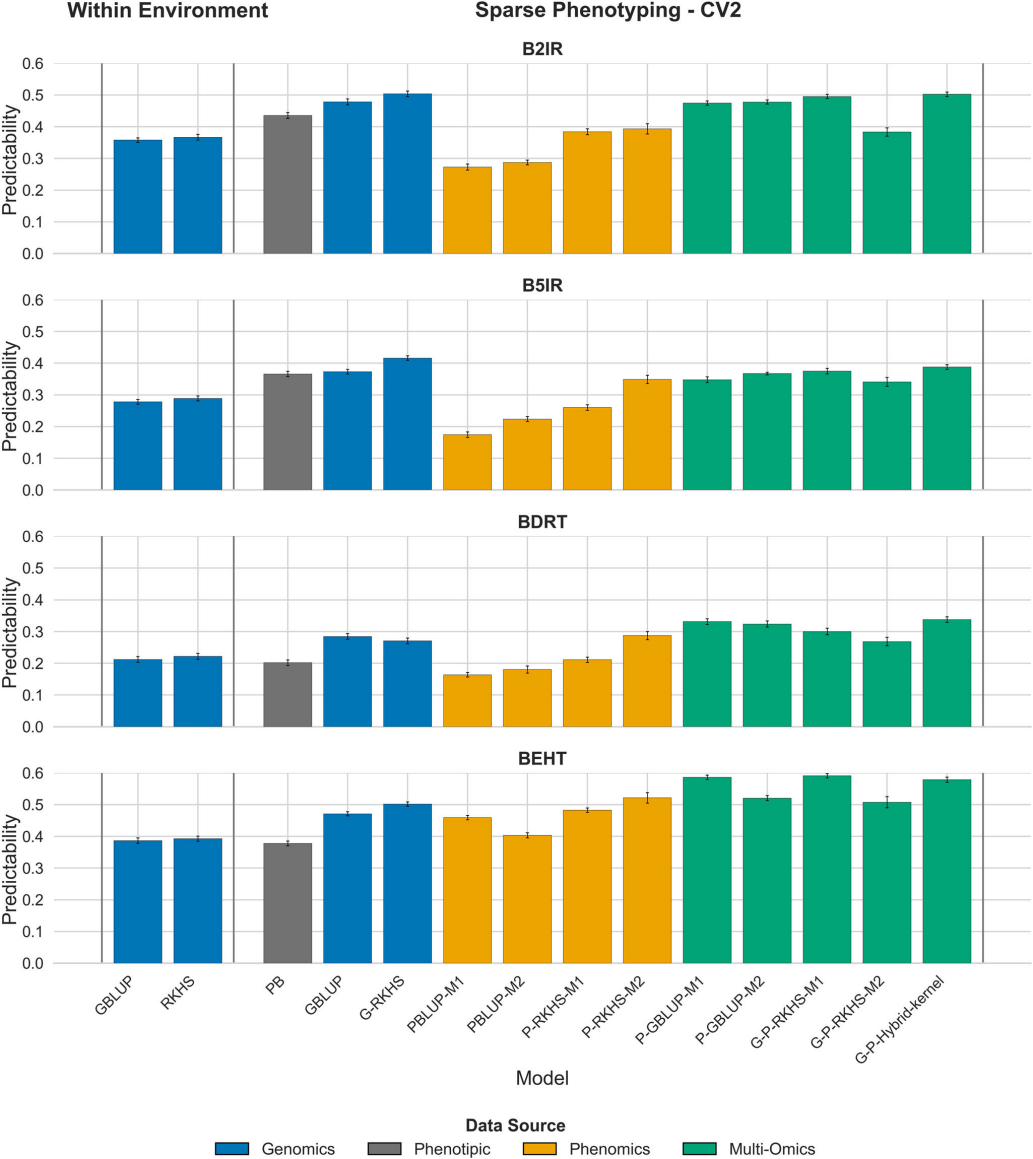
Model predictability in within‐environment validation and in sparse phenotyping (CV2) validation using only phenotypic, genomics‐based models, only phenomics‐based models, and combining genomics and phenomics. GBLUP, genomic best linear unbiased predictors; M1, method 1; M2, method 2; PB, phenotypic baseline; PBLUP, phenomic BLUP; P‐RKHS, phenomic RKHS; RKHS, reproducing kernel Hilbert space.

Genomics‐based model PA results in within‐environment validation using 10‐cycle 5‐fold cross‐validation are reported in Figure 6. GBLUP and RKHS showed no significant difference in PA. By contrast, differences in PA were visible across environments. Indeed, model PA ranged between 0.21 in BDRT (GBLUP) and 0.39 in BEHT (GBLUP and RKHS).

Figure 6 also shows the results of model PA in the sparse phenotyping design (CV2‐like validation) using only phenotypic, genomic, and phenomic data in M1 and M2, and combining both omics in M1 and M2.

Regarding only genomics‐based models, as expected, the differences between the within‐environment results and in a sparse phenotyping approach were evident. Indeed, PA in B2IR increased from 0.36 (GBLUP, within‐environment) to 0.48 (GBLUP, sparse phenotyping approach). In this strategy, the differences between the two models appeared more significant. For example, in the B5IR environment, PA ranged from 0.37 to 0.42 for GBLUP and RKHS, respectively. In addition, the PB model underperformed the genomics‐based models in all the simulated environments in the sparse phenotyping scenario. However, the PB model clearly outperformed the phenomics‐based models only in the B2IR environment, whereas it consistently performed worse than the multiomic models.

For models based solely on phenomic data, PA was generally lower than for models based solely on genomics. In this scenario, RKHS outperformed GBLUP in both M1 and M2. For example, PBLUP‐M2 showed a PA of 0.18 in BDRT, while P‐RKHS‐M2 showed a PA of 0.29 for the same environment. For these models, it seemed also evident that the M1 outperformed the M1. For instance, P‐RKHS PA for B5IR improved from 0.26 to 0.35, shifting from M1 to M2. In this context, the P‐RKHS‐M2 model consistently performed best across all environments.

Finally, models combining genomic and phenomic information generally achieved competitive PA compared to genomics‐only–based models among all the models evaluated, but the advantage of integration was environment‐dependent. For instance, using the G‐P‐RKHS‐M1 model, a PA for BEHT of 0.59 was achieved, whereas using only genomics PA showed only 0.50 (G‐RKHS). Interestingly, the hybrid kernel method showed marginal improvement compared to the other models that leveraged both omics in B2IR, B5IR, and BDRT. When genomics and phenomics were combined, the M2 method showed instability in this context, performing among the worst models (see G‐P‐RKHS‐M2).

## 4 DISCUSSION

This study evaluated the potential of genomics, phenomics, and their integration to improve GY prediction in elite bread wheat lines under a real‐world sparse phenotyping scenario. Using a large population evaluated across contrasting water and heat stress environments at CIMMYT, we demonstrated that PA can be significantly enhanced by leveraging multienvironment information and by integrating UAV‐based multispectral phenotyping with genomic data. Collectively, the results suggest that sparse phenotyping strategies combined with multiomic prediction models could be a practical and scalable solution for modern wheat breeding programs.

### 4.1 Genomic prediction and environmental dependency

PA using genomics alone showed clear variation across environments, with higher PA in more favorable or structured conditions (B5IR and BEHT) and lower PA under severe drought stress (BDRT). This pattern is consistent with previous studies reporting that the PA of genomic models for GY is highly environment‐dependent and tends to decline under extreme stress, where environmental variance and unmodeled physiological processes dominate genetic signal (Burgueño et al., 2012; Lopez‐Cruz et al., 2015). The moderate heritability estimates observed across environments further explain these differences and reinforce the need to model G × E interaction explicitly.

Within environments, GBLUP and RKHS performed similarly, suggesting that additive genomic models capture most of the exploitable signal when prediction is restricted to a single environment. Similar conclusions have been reported in wheat and other cereals, where nonlinear kernel methods do not consistently outperform linear models unless strong nonlinear responses or complex interaction patterns are present (Gianola & Van Kaam, 2008; Heslot et al., 2014). However, this apparent equivalence changes markedly under sparse phenotyping and cross‐environment prediction, highlighting the importance of model choice in applied breeding contexts.

### 4.2 Sparse phenotyping and cross‐environment information borrowing

One of the most relevant findings of this study is the clear advantage of sparse phenotyping designs when combined with multienvironment genomic prediction. Across all environments, PA under sparse phenotyping equaled or exceeded that obtained under within‐environment prediction, despite a substantial reduction in phenotypic observations (Figure 6). This result is in strong agreement with previous work demonstrating that genome‐enabled sparse testing improves PA by allowing models to borrow information across correlated environments (Crespo‐Herrera et al., 2021a).

The gains observed under sparse phenotyping were particularly pronounced in environments with lower within‐environment PA, such as B2IR and BDRT. This suggests that sparse phenotyping not only reduces phenotyping costs but also stabilizes predictions in challenging environments by exploiting shared genetic signal across trials. Genomics‐based models consistently outperformed the multienvironment PB model across all the tested environments. From a breeding program perspective, these results support the strategy of increasing the number of environments while reducing the number of genotypes per environment, provided that genomic data are available to connect trials.

### 4.3 Phenomics‐based prediction and spectral information

Correlation analyses further illustrate the complexity of spectral–yield relationships (Figure 4). While some wavelengths, particularly NIR, showed moderate positive associations with GY in stress environments, these relationships were weak or inconsistent under optimal conditions. This variability underscores a key limitation of phenomics: spectral signals are strongly context‐dependent and should not be expected to generalize uniformly across environments or developmental stages without appropriate modeling strategies. Additionally, for GY, SREG analysis revealed significant G × E interactions across all wavelengths derived from the UAV‐derived image processing (Figure 5).

In general, models based exclusively on phenomic data showed lower PA than genomic models, yet still captured meaningful information about GY. This is consistent with previous studies indicating that spectral reflectance traits are heritable and genetically structured, yet often insufficient on their own to fully predict complex traits such as yield (Krause et al., 2019). Additionally, phenomics‐based models showed lower performance than the PB in B2IR and B5IR but outperformed the PB in BEHT and BDRT. In phenomics‐only models, RKHS consistently outperformed GBLUP, indicating that nonlinear relationships between spectral reflectance and GY are important, particularly under stress conditions.

The comparison between the two phenomic strategies (M1 and M2) provides important methodological insights. Models based on M2, which predict missing wavelengths within each environment before constructing phenomic relationship matrices, consistently outperformed those based on M1. This result suggests that preserving environment‐specific spectral patterns prior to cross‐environment integration is critical for effective phenomic prediction. Similar findings have been reported in recent phenomic prediction studies, in which environment‐aware handling of spectral data improved robustness and predictive performance (Robert et al., 2022; Winn et al., 2023).

### 4.4 Integration of genomics and phenomics

The strongest and most consistent prediction accuracies were obtained from models that combined genomic and phenomic information. Multiomic models outperformed single‐omics approaches and the PB across nearly all environments, confirming the complementary nature of genomics and phenomics. Genomic data capture stable additive genetic relationships, whereas phenomic data reflect realized plant performance, canopy development, and physiological responses under specific environmental conditions. Their integration can therefore provide a more comprehensive representation of the factors driving variation in GY, but the benefit is expected to depend on the target environment, growth stage, and strength of the association between spectral information and yield.

These results align with previous studies reporting improved PA when genomic and spectral data are combined, particularly for biomass and yield‐related traits (Brault et al., 2022; Galán et al., 2020). The benefits of multiomic models were especially evident in stress‐prone environments such as BEHT, where phenomic data likely captured heat‐responsive canopy traits not fully explained by molecular markers alone.

The hybrid kernel approach showed marginal yet consistent improvements over standard multiomic models across several environments. Although the magnitude of these gains was modest, they suggest that explicitly modeling interactions between genomic and phenomic kernels can extract additional predictive signal. Similar incremental improvements have been reported in recent studies exploring hybrid or interaction‐based kernels for multiomic prediction (Cuevas et al., 2025). In applied breeding programs, even small gains in PA can translate into meaningful increases in genetic gain, particularly at advanced selection stages.

Similarly to our finding, the hybrid kernel approach was successfully tested using multiyear data from the winter wheat breeding program at Washington State University (Montesinos‐López et al., 2026). The authors demonstrated improvements in GY PA of up to 30% using the hybrid kernel approach compared to the reference models.

### 4.5 Implications for breeding program design

From a practical breeding standpoint, the results of this study raised several important implications. First, sparse phenotyping should be viewed not only as a cost‐saving strategy but also as a performance‐enhancing approach when combined with appropriate prediction models. While a phenotype‐only model can also sufficiently estimate the performance of untested lines in a tested environment, genomics can offer a more precise estimate of the breeding values of the tested lines across optimal and stressed environments. Second, phenomics should not be considered a replacement for genomics, but rather a complementary data source that enhances prediction when integrated properly.

In large‐scale wheat breeding programs such as CIMMYT's, where hundreds to thousands of elite lines are evaluated across contrasting irrigation and stress regimes, the integration of genomics and UAV‐based phenomics within sparse phenotyping designs offers a scalable and operationally feasible pathway to improve selection decisions. Importantly, the approaches evaluated here rely on routinely collected multispectral data and standard genomic resources, increasing their potential for adoption in operational breeding pipelines.

### 4.6 Theoretical interpretation of model behavior

The differences observed among genomic, phenomic, and multiomic prediction models can be interpreted in terms of the nature of the information each model captures and how this information propagates under a sparse and multienvironment strategy. Genomic prediction models such as GBLUP primarily exploit additive genetic relationships that are stable across environments and generations. This stability explains their strong, consistent performance across environments and their ability to transfer information across trials when using sparse phenotyping designs. However, because these models rely on average genetic effects, their predictive power can be limited when environment‐specific physiological responses play a dominant role.

Kernel‐based genomic models, such as RKHS, extend this framework by allowing nonlinear relationships among genotypes, thereby capturing complex response surfaces arising from G × E interactions. While these nonlinear effects may not be strongly expressed within a single environment, they become increasingly relevant when information is shared across heterogeneous environments, as observed under sparse phenotyping. This explains why RKHS‐based models tended to show advantages over linear models in cross‐environment prediction scenarios rather than in within‐environment analyses.

Phenomics‐based models operate under a fundamentally different principle. Instead of capturing inherited genetic relationships, phenomic relationship matrices summarize similarity in realized plant performance, which inherently reflects both genetic potential and environmental modulation. As a consequence, phenomic models are particularly sensitive to environment‐specific signals, such as canopy development and stress responses, but their predictive ability is reduced when such signals are weak or inconsistent across environments. The superior performance of nonlinear kernels in phenomics‐only models further indicates that the relationship between spectral reflectance and GY is inherently nonlinear and context‐dependent.

Multiomic models combine these complementary sources of information within a unified prediction framework. From a theoretical perspective, their improved performance stems from their ability to jointly model stable additive genetic effects and environment‐responsive phenotypic expression. Genomic kernels anchor predictions across environments by preserving genetic relatedness, while phenomic kernels modulate predictions by incorporating realized physiological responses. Hybrid kernel formulations further enable interactions among these information sources, allowing the model to weight genomic and phenomic contributions differently across environments. Although the resulting gains are often incremental, they reflect a more faithful representation of the biological processes underlying GY variation.

### 4.7 Limitations and future perspectives

Several limitations of this study should be acknowledged. The analysis was conducted within a single growing season and location, and extending the framework across years would allow the evaluation of temporal stability and robustness. Additionally, UAV flights were conducted on common calendar dates across managed environments rather than explicitly aligned by thermal time or physiological stage. Consequently, cross‐environment spectral differences may partly reflect differences in crop development and maturity, especially under drought and heat stress. Future studies should evaluate phenomic prediction using measurements aligned by thermal time, growth stage, or phenological landmarks. In addition, only multispectral data were considered; hyperspectral, thermal, or structural traits may capture additional physiological information relevant to stress tolerance. Future research should also explore dynamic modeling approaches that explicitly exploit time‐series phenomic data rather than aggregated measures. Additionally, artificial intelligence‐based strategies, especially deep learning, could better leverage multiple omics data sources than non‐parametric models such as RKHS (Crossa et al., 2025).

Finally, while this study focused on PA, future work should assess the impact of these strategies on realized genetic gain under selection. Integrating multiomic prediction models with selection indices and economic weights would further strengthen the link between predictive performance and breeding outcomes.

## 5 CONCLUSIONS

In summary, this study shows that integrating genomics and UAV‐based phenomics within clearly defined sparse‐testing designs can improve GY prediction in elite wheat lines across contrasting environments, although the benefit of phenomic integration is environment‐dependent. Genomic prediction provides a robust foundation, phenomics can add environment‐responsive information, and their integration may yield more accurate predictions when spectral traits capture relevant physiological variation. These findings support continued evaluation and targeted adoption of genomic‐phenomic sparse‐testing strategies in modern wheat breeding programs facing resource constraints and increasing environmental variability.

## AUTHOR CONTRIBUTIONS

**Guillermo Gerard**: Conceptualization; data curation; methodology; writing—review and editing. **Velu Govindan**: Data curation; methodology. **Susanne Dreisigacker**: Data curation; methodology. **Keith A Gardner**: Supervision. **Flavio Breseghello**: Supervision. **Leonardo Crespo**: Data curation; methodology. **Matthew Reynolds**: Methodology. **Jose Crossa**: Conceptualization; methodology; writing—original draft. **Paolo Vitale**: Conceptualization; data curation; formal analysis; methodology; writing—original draft.

## CONFLICT OF INTEREST STATEMENT

The authors declare no conflicts of interest.

## Supporting information



Supplementary Table S1 and Figures S1‐S4 are available in the online supplementary materials as a separate document (Supplementary_File.docx).

## Data Availability

The datasets generated and analyzed during this study, including best linear unbiased estimates (BLUEs) for grain yield and spectral wavelengths, as well as the corresponding genotypic information, are publicly available in the CIMMYT data repository (https://doi.org/10.71682/10549399).
