# Supplementary material for: Sparse phenotyping for wheat grain yield enabled by multiomics prediction
Source: Plant Genome. 2026 Sep 6;19(3):e70298. doi: 10.1002/tpg2.70298 (PMC13547598; doi:10.1002/tpg2.70298)
Supplement: Supplementary file 1 [file TPG2-19-e70298-s001.docx]

Table S1 Descriptive statistics of the best linear unbiased estimates (BLUEs), Variance Components, and heritability for the wavelengths in each flight date.

| **Environment** | **date** | **Trait** | **Min** | **Mean** | **Max** | **sd** | **CV** | **vg** | **ve** | **H2** |
| --- | --- | --- | --- | --- | --- | --- | --- | --- | --- | --- |
| B2IR | 2023.02.15 | Blue | 1.87E-02 | 2.30E-02 | 2.87E-02 | 1.68E-03 | 7.31E+00 | 2.29E-06 | 6.15E-07 | 8.82E-01 |
|  |  | Green | 3.09E-02 | 3.72E-02 | 4.52E-02 | 2.38E-03 | 6.41E+00 | 4.34E-06 | 1.54E-06 | 8.49E-01 |
|  |  | NIR | 1.72E-01 | 1.97E-01 | 2.26E-01 | 8.26E-03 | 4.20E+00 | 4.28E-05 | 2.84E-05 | 7.51E-01 |
|  |  | Red | 1.84E-02 | 2.27E-02 | 2.79E-02 | 1.51E-03 | 6.63E+00 | 1.31E-06 | 1.25E-06 | 6.78E-01 |
|  |  | RedEdge | 6.11E-02 | 7.01E-02 | 8.03E-02 | 3.31E-03 | 4.72E+00 | 7.91E-06 | 3.56E-06 | 8.16E-01 |
|  | 2023.02.23 | Blue | 1.86E-02 | 2.43E-02 | 3.08E-02 | 1.78E-03 | 7.35E+00 | 2.25E-06 | 1.09E-06 | 8.05E-01 |
|  |  | Green | 3.11E-02 | 3.83E-02 | 4.69E-02 | 2.50E-03 | 6.54E+00 | 3.94E-06 | 2.74E-06 | 7.42E-01 |
|  |  | NIR | 1.79E-01 | 2.04E-01 | 2.32E-01 | 8.35E-03 | 4.08E+00 | 4.79E-05 | 2.82E-05 | 7.73E-01 |
|  |  | Red | 1.85E-02 | 2.45E-02 | 3.38E-02 | 1.99E-03 | 8.12E+00 | 1.55E-06 | 2.90E-06 | 5.16E-01 |
|  |  | RedEdge | 6.14E-02 | 7.08E-02 | 8.16E-02 | 3.54E-03 | 5.00E+00 | 7.51E-06 | 6.22E-06 | 7.07E-01 |
|  | 2023.03.03 | Blue | 2.13E-02 | 2.65E-02 | 3.44E-02 | 1.85E-03 | 6.96E+00 | 1.87E-06 | 1.49E-06 | 7.16E-01 |
|  |  | Green | 3.23E-02 | 4.06E-02 | 5.09E-02 | 2.48E-03 | 6.09E+00 | 2.54E-06 | 3.72E-06 | 5.77E-01 |
|  |  | NIR | 1.87E-01 | 2.11E-01 | 2.38E-01 | 7.63E-03 | 3.62E+00 | 3.12E-05 | 3.67E-05 | 6.30E-01 |
|  |  | Red | 2.15E-02 | 2.73E-02 | 4.13E-02 | 2.48E-03 | 9.06E+00 | 1.68E-06 | 4.41E-06 | 4.33E-01 |
|  |  | RedEdge | 6.01E-02 | 7.17E-02 | 8.75E-02 | 3.23E-03 | 4.51E+00 | 4.21E-06 | 7.23E-06 | 5.38E-01 |
|  | 2023.03.14 | Blue | 2.41E-02 | 2.95E-02 | 3.52E-02 | 1.50E-03 | 5.10E+00 | 1.45E-06 | 9.57E-07 | 7.52E-01 |
|  |  | Green | 3.78E-02 | 4.49E-02 | 5.27E-02 | 2.14E-03 | 4.76E+00 | 2.51E-06 | 2.33E-06 | 6.83E-01 |
|  |  | NIR | 1.49E-01 | 1.66E-01 | 2.01E-01 | 6.82E-03 | 4.10E+00 | 2.03E-05 | 3.56E-05 | 5.33E-01 |
|  |  | Red | 2.93E-02 | 3.60E-02 | 4.48E-02 | 2.31E-03 | 6.42E+00 | 3.10E-06 | 2.43E-06 | 7.18E-01 |
|  |  | RedEdge | 6.78E-02 | 7.60E-02 | 8.75E-02 | 2.93E-03 | 3.86E+00 | 4.00E-06 | 5.31E-06 | 6.01E-01 |
|  | 2023.03.30 | Blue | 3.77E-02 | 5.13E-02 | 6.44E-02 | 4.13E-03 | 8.05E+00 | 1.17E-05 | 5.24E-06 | 8.17E-01 |
|  |  | Green | 6.34E-02 | 8.63E-02 | 1.05E-01 | 6.17E-03 | 7.14E+00 | 2.61E-05 | 1.26E-05 | 8.05E-01 |
|  |  | NIR | 1.71E-01 | 1.95E-01 | 2.21E-01 | 7.09E-03 | 3.64E+00 | 2.81E-05 | 2.80E-05 | 6.68E-01 |
|  |  | Red | 6.56E-02 | 1.02E-01 | 1.31E-01 | 1.18E-02 | 1.16E+01 | 1.00E-04 | 3.17E-05 | 8.64E-01 |
|  |  | RedEdge | 1.06E-01 | 1.41E-01 | 1.67E-01 | 9.15E-03 | 6.50E+00 | 5.85E-05 | 2.43E-05 | 8.28E-01 |
| B5IR | 2023.02.15 | Blue | 0.017044 | 0.024601 | 0.031098 | 0.00225 | 9.14661 | 4.38E-06 | 7.93E-07 | 0.916927 |
|  |  | Green | 0.029467 | 0.042122 | 0.050315 | 0.003423 | 8.125657 | 1.01E-05 | 2.09E-06 | 0.906045 |
|  |  | NIR | 0.181011 | 0.224451 | 0.263518 | 0.011847 | 5.278362 | 8.42E-05 | 5.83E-05 | 0.742926 |
|  |  | Red | 0.016261 | 0.02252 | 0.029064 | 0.001908 | 8.47068 | 2.42E-06 | 1.48E-06 | 0.765313 |
|  |  | RedEdge | 0.061019 | 0.079713 | 0.094128 | 0.005206 | 6.531228 | 2.22E-05 | 5.58E-06 | 0.888487 |
|  | 2023.02.23 | Blue | 0.018867 | 0.027475 | 0.037093 | 0.003069 | 11.16852 | 8.42E-06 | 1.14E-06 | 0.936431 |
|  |  | Green | 0.030803 | 0.045198 | 0.056926 | 0.003974 | 8.791701 | 1.36E-05 | 2.60E-06 | 0.912542 |
|  |  | NIR | 0.204235 | 0.246903 | 0.285371 | 0.013437 | 5.442322 | 0.000112 | 7.20E-05 | 0.757641 |
|  |  | Red | 0.016812 | 0.023776 | 0.030717 | 0.002298 | 9.663776 | 4.41E-06 | 1.03E-06 | 0.895088 |
|  |  | RedEdge | 0.061913 | 0.083538 | 0.100446 | 0.005528 | 6.617713 | 2.53E-05 | 6.09E-06 | 0.892556 |
|  | 2023.03.03 | Blue | 0.018751 | 0.024665 | 0.034907 | 0.002566 | 10.40417 | 5.33E-06 | 1.27E-06 | 0.893563 |
|  |  | Green | 0.02959 | 0.040775 | 0.052239 | 0.003457 | 8.477144 | 9.13E-06 | 2.93E-06 | 0.861567 |
|  |  | NIR | 0.206433 | 0.241685 | 0.278524 | 0.013438 | 5.559997 | 0.00011 | 7.26E-05 | 0.751876 |
|  |  | Red | 0.016788 | 0.022158 | 0.030027 | 0.002001 | 9.030552 | 2.56E-06 | 1.63E-06 | 0.758571 |
|  |  | RedEdge | 0.058742 | 0.075087 | 0.09143 | 0.005102 | 6.794569 | 1.94E-05 | 6.93E-06 | 0.84844 |
|  | 2023.03.14 | Blue | 0.021278 | 0.025988 | 0.032379 | 0.001925 | 7.406797 | 2.57E-06 | 9.94E-07 | 0.838264 |
|  |  | Green | 0.032605 | 0.039837 | 0.049285 | 0.002648 | 6.647193 | 4.67E-06 | 2.26E-06 | 0.804987 |
|  |  | NIR | 0.16564 | 0.196135 | 0.230011 | 0.010838 | 5.525813 | 6.09E-05 | 6.37E-05 | 0.656646 |
|  |  | Red | 0.021704 | 0.026439 | 0.033263 | 0.00176 | 6.65558 | 1.64E-06 | 1.38E-06 | 0.703545 |
|  |  | RedEdge | 0.057966 | 0.068237 | 0.08021 | 0.003412 | 5.00029 | 7.01E-06 | 4.96E-06 | 0.738499 |
|  | 2023.03.30 | Blue | 0.024912 | 0.030679 | 0.038267 | 0.002084 | 6.792374 | 2.69E-06 | 1.93E-06 | 0.73643 |
|  |  | Green | 0.04271 | 0.053663 | 0.074845 | 0.003573 | 6.659114 | 8.11E-06 | 5.39E-06 | 0.750534 |
|  |  | NIR | 0.181235 | 0.216232 | 0.25523 | 0.011402 | 5.272929 | 7.02E-05 | 6.16E-05 | 0.695073 |
|  |  | Red | 0.027778 | 0.03676 | 0.065382 | 0.003524 | 9.586895 | 7.38E-06 | 5.32E-06 | 0.734949 |
|  |  | RedEdge | 0.073142 | 0.087705 | 0.118362 | 0.004657 | 5.31041 | 1.35E-05 | 9.88E-06 | 0.732182 |
| BDRT | 2023.02.15 | Blue | 0.022823 | 0.026879 | 0.033647 | 0.001473 | 5.48054 | 1.51E-06 | 8.07E-07 | 0.788799 |
|  |  | Green | 0.037021 | 0.042435 | 0.050275 | 0.002087 | 4.918196 | 2.75E-06 | 1.80E-06 | 0.753334 |
|  |  | NIR | 0.142138 | 0.165842 | 0.185149 | 0.006067 | 3.658112 | 2.45E-05 | 1.60E-05 | 0.753924 |
|  |  | Red | 0.025956 | 0.031716 | 0.047915 | 0.002264 | 7.137736 | 1.90E-06 | 4.49E-06 | 0.458238 |
|  |  | RedEdge | 0.065901 | 0.073479 | 0.0821 | 0.002616 | 3.560804 | 4.30E-06 | 3.01E-06 | 0.740646 |
|  | 2023.02.23 | Blue | 0.023275 | 0.029196 | 0.036307 | 0.001927 | 6.599831 | 2.07E-06 | 1.97E-06 | 0.676929 |
|  |  | Green | 0.037382 | 0.045079 | 0.052759 | 0.002594 | 5.75333 | 3.27E-06 | 4.22E-06 | 0.607746 |
|  |  | NIR | 0.149525 | 0.180082 | 0.210234 | 0.009008 | 5.001926 | 3.97E-05 | 4.94E-05 | 0.61635 |
|  |  | Red | 0.02598 | 0.035454 | 0.050974 | 0.003472 | 9.793116 | 3.72E-06 | 9.94E-06 | 0.427787 |
|  |  | RedEdge | 0.068184 | 0.078248 | 0.087202 | 0.003424 | 4.375333 | 5.06E-06 | 8.63E-06 | 0.539555 |
|  | 2023.03.03 | Blue | 0.028366 | 0.034243 | 0.041772 | 0.001998 | 5.834896 | 2.08E-06 | 2.65E-06 | 0.611345 |
|  |  | Green | 0.043938 | 0.052553 | 0.062958 | 0.002858 | 5.43853 | 3.78E-06 | 5.84E-06 | 0.564017 |
|  |  | NIR | 0.150819 | 0.183415 | 0.212451 | 0.009367 | 5.106972 | 4.55E-05 | 4.80E-05 | 0.655032 |
|  |  | Red | 0.033849 | 0.045546 | 0.064589 | 0.004122 | 9.051131 | 5.81E-06 | 1.37E-05 | 0.459134 |
|  |  | RedEdge | 0.077223 | 0.088042 | 0.099135 | 0.003789 | 4.303267 | 5.83E-06 | 1.14E-05 | 0.506581 |
|  | 2023.03.14 | Blue | 0.039982 | 0.046472 | 0.056336 | 0.00261 | 5.616791 | 3.03E-06 | 4.38E-06 | 0.580355 |
|  |  | Green | 0.060265 | 0.072548 | 0.089469 | 0.004232 | 5.833581 | 7.75E-06 | 1.22E-05 | 0.559115 |
|  |  | NIR | 0.130043 | 0.154659 | 0.180544 | 0.008234 | 5.324033 | 2.77E-05 | 4.62E-05 | 0.545124 |
|  |  | Red | 0.067142 | 0.084289 | 0.114817 | 0.006361 | 7.546473 | 2.01E-05 | 2.28E-05 | 0.637259 |
|  |  | RedEdge | 0.099736 | 0.119172 | 0.145364 | 0.006217 | 5.217231 | 1.54E-05 | 2.89E-05 | 0.515448 |
|  | 2023.03.30 | Blue | 0.057982 | 0.073037 | 0.086824 | 0.004587 | 6.280758 | 1.21E-05 | 1.12E-05 | 0.683865 |
|  |  | Green | 0.090574 | 0.111852 | 0.130585 | 0.006501 | 5.81243 | 2.17E-05 | 2.70E-05 | 0.617181 |
|  |  | NIR | 0.156878 | 0.186472 | 0.213518 | 0.008522 | 4.570022 | 3.02E-05 | 6.07E-05 | 0.498868 |
|  |  | Red | 0.137939 | 0.16384 | 0.190075 | 0.008363 | 5.104336 | 3.12E-05 | 5.22E-05 | 0.544857 |
|  |  | RedEdge | 0.14583 | 0.172285 | 0.195857 | 0.008092 | 4.696762 | 2.68E-05 | 5.54E-05 | 0.491201 |
| BEHT | 2023.02.15 | Blue | 0.017714 | 0.022778 | 0.028101 | 0.001516 | 6.654792 | 1.68E-06 | 6.55E-07 | 0.836953 |
|  |  | Green | 0.028431 | 0.036042 | 0.042358 | 0.002053 | 5.696711 | 2.47E-06 | 1.93E-06 | 0.719426 |
|  |  | NIR | 0.018255 | 0.022937 | 0.030702 | 0.001672 | 7.288131 | 1.21E-06 | 2.07E-06 | 0.538711 |
|  |  | Red | 0.171252 | 0.195846 | 0.225731 | 0.008207 | 4.190444 | 4.58E-05 | 2.48E-05 | 0.786561 |
|  |  | RedEdge | 0.055537 | 0.065231 | 0.075878 | 0.00285 | 4.369193 | 4.55E-06 | 3.98E-06 | 0.695366 |
|  | 2023.02.23 | Blue | 0.018891 | 0.024021 | 0.029904 | 0.001624 | 6.760674 | 1.73E-06 | 9.73E-07 | 0.780576 |
|  |  | Green | 0.031529 | 0.038481 | 0.045871 | 0.002276 | 5.914039 | 2.62E-06 | 2.82E-06 | 0.65033 |
|  |  | NIR | 0.018892 | 0.024964 | 0.033369 | 0.002272 | 9.101568 | 2.19E-06 | 3.14E-06 | 0.582367 |
|  |  | Red | 0.172372 | 0.195682 | 0.219396 | 0.007536 | 3.851332 | 3.70E-05 | 2.25E-05 | 0.767299 |
|  |  | RedEdge | 0.059832 | 0.068469 | 0.077819 | 0.00289 | 4.22086 | 3.56E-06 | 5.64E-06 | 0.558441 |
|  | 2023.03.03 | Blue | 0.01947 | 0.02454 | 0.031086 | 0.001734 | 7.0642 | 1.84E-06 | 1.38E-06 | 0.728126 |
|  |  | Green | 0.030764 | 0.038641 | 0.047111 | 0.002473 | 6.399842 | 3.29E-06 | 3.38E-06 | 0.660434 |
|  |  | NIR | 0.019846 | 0.026564 | 0.038558 | 0.002387 | 8.985759 | 2.67E-06 | 3.71E-06 | 0.590285 |
|  |  | Red | 0.159561 | 0.190326 | 0.216204 | 0.008432 | 4.430209 | 4.15E-05 | 3.81E-05 | 0.684904 |
|  |  | RedEdge | 0.05663 | 0.067004 | 0.077695 | 0.003241 | 4.836809 | 4.98E-06 | 6.85E-06 | 0.592817 |
|  | 2023.03.14 | Blue | 0.022218 | 0.027692 | 0.035121 | 0.002209 | 7.978184 | 3.48E-06 | 1.50E-06 | 0.822807 |
|  |  | Green | 0.035267 | 0.045537 | 0.062582 | 0.003896 | 8.554937 | 1.05E-05 | 4.83E-06 | 0.813621 |
|  |  | NIR | 0.025505 | 0.035764 | 0.059515 | 0.004274 | 11.95099 | 1.39E-05 | 3.57E-06 | 0.886255 |
|  |  | Red | 0.130502 | 0.157267 | 0.184137 | 0.008401 | 5.341731 | 3.67E-05 | 5.40E-05 | 0.575852 |
|  |  | RedEdge | 0.061047 | 0.076013 | 0.102068 | 0.005485 | 7.215469 | 2.01E-05 | 1.01E-05 | 0.798817 |
|  | 2023.03.30 | Blue | 0.032307 | 0.046276 | 0.059426 | 0.00387 | 8.362949 | 1.15E-05 | 3.90E-06 | 0.854945 |
|  |  | Green | 0.054057 | 0.078623 | 0.094947 | 0.005105 | 6.492626 | 1.77E-05 | 9.97E-06 | 0.780599 |
|  |  | NIR | 0.059177 | 0.089749 | 0.118168 | 0.010175 | 11.33707 | 8.19E-05 | 2.11E-05 | 0.885699 |
|  |  | Red | 0.132599 | 0.167827 | 0.206626 | 0.011139 | 6.6373 | 8.93E-05 | 3.54E-05 | 0.834502 |
|  |  | RedEdge | 0.091596 | 0.123757 | 0.142365 | 0.006291 | 5.083493 | 2.39E-05 | 1.93E-05 | 0.711515 |

Figure S1 Phenotypic correlation among the wavelengths collected at every flight and grain yield for the B2IR environment.

Figure S2 Phenotypic correlation among the wavelengths collected at every flight and grain yield for the BEHT environment.

Figure S3 Phenotypic correlation among the wavelengths collected at every flight and grain yield for the BDRT environment.

Figure S4 Phenotypic correlation among the wavelengths collected at every flight and grain yield for the B5IR environment.
